# Innovations of a Learning Community to Optimize Dementia-Capable Adult Day Services

**DOI:** 10.1093/geroni/igaf122.1711

**Published:** 2025-12-31

**Authors:** Orly Tonkikh, Jessica Famula, Janice Bell, Shikha Bhurtel, Quynh Vo, Heather Young

**Affiliations:** University of Haifa, Haifa, HaZafon, Israel; University of California, Davis, Davis, California, United States; Betty Irene Moore School Of Nursing at UC Davis, Sacramento, California, United States; University of California, Davis, Davis, California, United States; University of California, Davis, Davis, California, United States; Betty Irene Moore School Of Nursing at UC Davis, Sacramento, California, United States

## Abstract

Adult day services (ADS) offer social and healthcare services to enhance quality of life of persons with Alzheimer’s disease and related dementias (ADRD) and their family caregivers. Sustainable approaches to standardized dementia-capable ADS are vital. This study describes the structures, processes and accomplishments of a learning community developed to advance and integrate best practices for dementia-capable ADS as part of Cal-COMPASS (California Community Program for Alzheimer’s Services and Supports) program, involving seven sites. We collected learning community documentation and conducted 14 focus groups with 16-24 staff and directors from each organization participating in Cal-COMPASS at baseline and upon completion. The data were analyzed using qualitative descriptive methods. To develop an optimal dementia-capable ADS model, five workgroups focused on structural and process-related elements required for effective dementia-capable ADS including staffing and professional training, financing and sustainability, screening and measurement, and caregiver support practices. The learning community developed a comprehensive set of guidelines and resources to promote the standardization and sustainment of dementia-capable services and to make them accessible to the diverse populations of persons with ADRD and their care partners in California. Each workgroup had accomplishments, including recommendations for assessment and care. The participants identified substantial long-term expertise, a wide range of skill, strength, and capacity mix as facilitators of collaborative knowledge sharing and action. Future research could explore how learning communities promote quality of care, as well as long-term adoption and impact of the established practices for persons with ADRD and their caregivers.

